# Multi-Analytical Characterization and Radiocarbon Dating of a Roman Egyptian Mummy Portrait

**DOI:** 10.3390/molecules26175268

**Published:** 2021-08-30

**Authors:** Alice Dal Fovo, Mariaelena Fedi, Gaia Federico, Lucia Liccioli, Serena Barone, Raffaella Fontana

**Affiliations:** 1 National Research Council—National Institute of Optics, Largo E. Fermi 6, 50125 Firenze, Italy; raffaella.fontana@ino.cnr.it; 2 National Institute of Nuclear Physics, Via Bruno Rossi 1, Sesto Fiorentino, 50019 Firenze, Italy; fedi@fi.infn.it (M.F.); liccioli@fi.infn.it (L.L.); serena.barone@fi.infn.it (S.B.); 3 OPD-Scuola di Alta Formazione e Studio, Via Alfani 78, 50121 Firenze, Italy; gaiafederico91@gmail.com

**Keywords:** Fayum mummy portraits, radiocarbon dating, encaustic paint, multispectral reflectography, microprofilometry, fibre-optics reflectance spectroscopy, optical coherence tomography

## Abstract

Fayum mummy portraits, painted around 2000 years ago, represent a fascinating fusion of Egyptian and Graeco-Roman funerary and artistic traditions. Examination of these artworks may provide insight into the Roman Empire’s trade and economic and social structure during one of its most crucial yet still hazy times of transition. The lack of proper archaeological documentation of the numerous excavated portraits currently prevents their chronological dating, be it absolute or relative. So far, their production period has been defined essentially on the basis of the relevant differences in their pictorial style. Our study introduces the use of Accelerator Mass Spectrometry (AMS) to assess the age of a fragment of an encaustic painting belonging to the corpus of the Fayum portraits. The unexpected age resulting from ^14^C analysis suggests the need to reconsider previous assumptions regarding the period of production of the Fayum corpus. Furthermore, our multi-analytical, non-invasive approach yields further details regarding the fragment’s pictorial technique and constituting materials, based on spectral and morphological analysis and cross-sectional examination.

## 1. Introduction

The Graeco-Roman funerary painted artworks known as the corpus of Fayum mummy portraits, discovered in the area surrounding the Fayum Oasis on the west bank of the Nile River, displays a unique array of ancient Egyptian and Graeco-Roman cultural influences [1,2,3,4,5,6]. These depictions of real-life human faces, with their lifelike expressions and highly individualized traits, belong to the world’s oldest portrait-painting tradition in known history, believed to date back to the Roman rule in Egypt (30 BC–4th century AD) [7,8]. The resulting cultural immingling is witnessed in a combination of funerary traditions, bringing together Egyptian embalming practices and Roman burgeoning experimentation with portraiture, namely faithful painted representations of the deceased’s face to be placed on the latter’s mummified body. These frontal and three-quarters portraits were painted using a naturalistic style, clearly influenced by the Hellenistic tradition present in Egypt since the start of Macedonian domination (4th century BC). Funerary portraits could be painted directly on the linen shroud wrapping the deceased, or on wooden tablets that were either placed on the sarcophagus or inserted between the mummy’s bandages [9]. Of the approximately 1000 extant portraits currently scattered throughout the world, the provenance of many remains untraced, as most of them have been illicitly excavated from their first discovery in 1615 by P. Della Valle onwards, and then sold on the antiquarian market with poor or absent documentation on their origin. Only after 1890, with the excavation campaign by W. F. Petri [10], the archaeological findings of Fayum were systematically documented. Funerary portraits were found not only in the Fayum Oasis, but also along the Nile valley, at Saqqara (south of Cairo), Deir el-Bahari (west bank across Luxor), Panopolis (now Achmim), and Antinoopolis (now Sheikh ‘Ibada) [11].

In recent years, a comprehensive study carried out within the framework of the APPEAR Project [12] has led to the examination of 61 mummy paintings (51 mummy portraits and 10 panel paintings) from fourteen museums to gain a better understanding of the materials and techniques used to manufacture them [13]. The results showed that most of the paintings were realized with either encaustic (using beeswax as a binding medium for the pigments), or tempera paint (containing predominantly animal glue)—or a combination of both. The use of tempera and encaustic required both knowledge and mastery of the media, thus suggesting that the relevant technique was likely passed on from one artist to another in a studio setting [13].

The textile fibre usually found in Egyptian funeral paraphernalia, and, more specifically, in painted burial cloths, is linen [14]. Before painting, the textile support was often prepared with a primer made of sulphate or calcium carbonate, used to fill the discontinuities of the fabric, making its surface flat and smooth. A layer of wax or animal glue could also be applied to prepare for the drawing [3,4]. Preparatory sketches were commonly drawn with a brush, with grey, black, and sometimes red marks. Shadows were also defined at this stage with more diluted brushstrokes of the same hue [15]. For encaustic painting, beeswax was melted and mixed with powdered pigments. Typically, a bronze or iron tool, called cauterium in Pliny’s *Naturalis Historia* [16], was used to unify the brushstrokes once the painting had solidified, thus obtaining the characteristic smooth and shiny aspect marks on the surface [5]. Pigments were almost always naturally occurring earth colours, as it was customary throughout the Pharaonic period, although there is evidence that the Romans introduced some innovations, notably red lead, and the use of madder [15].

In Graeco-Roman colonies such as Antinoopolis, painted portraits were only found on the mummies of affluent individuals, and were probably a way to emphasize their social status and affiliation to the Empire, as witnessed by the clear influence of imperial fashion in the depicted hairstyles, clothing, and jewellery. Later portraits display stylistic signs that may indicate an affinity with the Christian doctrine and are consistent with the latter’s increasing presence within the Roman Empire. Evidence suggests that the use of funerary portraits dramatically decreased around the first half of the third century AD, a time characterised by a gradual weakening of Roman authority and the abandonment of the Egyptian traditional ritual of mummification [17]. The most recent portraits are traditionally dated to the end of the 3rd century AD and were found exclusively in the city of Antinoopolis [3,4]. They may be considered the turning point in the transition from the classical Greek naturalistic style to the new Paleo-Christian iconographic language that would find its full-fledged expression in Byzantine art.

So far, funerary portraits have been dated on the basis of stylistic differences in their pictorial style: while some display a high level of accuracy and naturalism in the physiognomic rendering of faces, others are characterised by a more schematic drawing technique, which appears to follow a repetitive pattern. Naturalistic portraits were believed to be the most ancient in the corpus, i.e., probably painted by Greek artists that had migrated to the Egyptian colonies following the Alexandrian conquest. The progressive loss of knowledge and skills among the new generations of artists would therefore be the reason behind the gradual shift towards a more schematic and pattern-like painting style [18]. There are scholars, however, who disagree with this interpretation, arguing that schematic and naturalistic portraits share numerous similar details, which are easily ascribable to the fashion in vogue throughout the Empire during a specific timeframe [1]. Following this second line of reasoning, different painting styles and features coexisting in portraits would be indicators of the different geographical areas of their production, rather than their age. Neither interpretation, however, effectively addresses the dating issue of the individual portraits, since no corroborating evidence was ever collected at the time of the portraits’ improper excavation and illicit distribution, and, as far as our knowledge, no scientific method has ever been applied to assess the age of the paintings.

In this study, an encaustic painted fragment of funerary linen shroud belonging to the corpus of the Fayum portraits was analysed and dated with scientific methods for the first time. The fragment was found in the southern city of Antinoopolis and is part of the archaeological collection of the G. Vitelli Papyrological Institute of Florence. Analyses were carried out at the Opificio delle Pietre Dure (Florence) as part of the portrait’s restoration campaign, which allowed for a direct correlation of the acquired data and information provided by the conservator on the painting technique and constituting materials. Visible-Near infrared (Vis-NIR) multispectral reflectography and Fibre-Optics Reflectance Spectroscopy (FORS) enabled the identification and mapping of the main pigments used, as well as the unveiling of details not perceivable to the naked eye. Laser scanning profilometry was used to carry out micrometric morphological measurements of the painting’s surface, to highlight features related to the pictorial technique. Spectral-domain Optical Coherence Tomography (Sd-OCT) was performed to obtain cross-sectional data on the stratigraphy of the paint layers in a non-invasive way. Finally, the age of the portrait was assessed using radiocarbon dating by Accelerator Mass Spectrometry (AMS).

## 2. Materials and Methods

### 2.1. The Case Study

**Figure 1:**
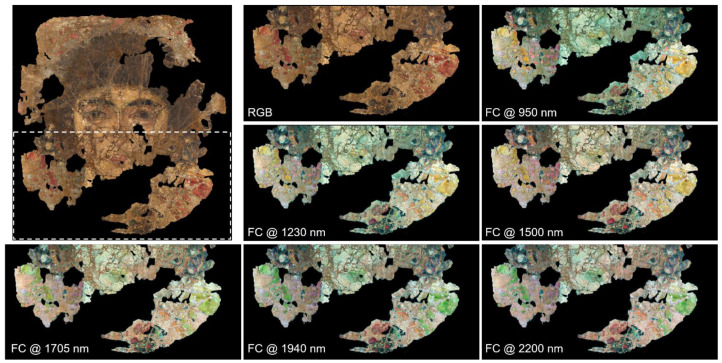
Colour image of the Fayum mummy portrait highlighting the area (dashed white rectangle) showed in the false colour NRG images at different wavelengths.

The encaustic painting analysed in this study is a fragment of a funerary portrait depicting a young woman (Figure 1, top left). Conservation data only documents the artwork’s history starting from its excavation in 1965 [19], while nothing is known about its previous existence. At the time of excavation, the fragment was not intact but broken into two separate pieces, which were placed in a box together with other pieces of fabrics without any particular precaution. The box was stored in the excavation camp until it was transported by ship to Naples, finally reaching the repository of the National Archaeological Museum of Florence, where it has been stored for approximately fifty years without ever being opened. When the restoration campaign was launched, the fragment was found crumpled and covered in dust. The paint layer appeared extremely patchy and crackled, especially on the skin tone, and there were visible abrasions along with the textile deformations, where the colour had detached. Initial cleaning improved the readability of the figure, uncovering large, well-defined dark eyes, typical of the Graeco-Roman portrait tradition. Touches of light on the cheekbones enhance the three-dimensionality of the woman’s features, outlined by a fine contour line. Her face is framed by thick brown hair gathered in an elaborate hairdo, which, however, is not easy to read due to the consistent loss of paint. She seems to be wearing jewellery, as shown by the presence of a red decoration (possibly a hoop earring of considerable size). A line of yellowish pearls against a dark background may indicate that she is wearing jewels in her hair too, denoting her high socioeconomic status. Red brushstrokes in the bottom-right portion of the portrait may be attributed to the neckline of the dress. Traces of light bluish colour, possibly belonging to the background, are visible at the bottom-right corner, near her neck, while a yellow-red background is distinguishable at the top of the fragment, behind the woman’s head.

### 2.2. Multispectral Reflectography and Fibre-Optics Reflectance Spectroscopy (FORS)

The multispectral scanner developed at the National Research Council–National Institute of Optics (CNR-INO) allows for the simultaneous acquisition of 32 narrow-band images (16 VIS and 16 NIR images) through whiskbroom scanning in the range of 390–2500 nm [20,21]. A square-shaped fibre bundle collects the reflected light from a single point of the scanned surface and delivers it to a set of Si and InGaAs photodiodes, each of them equipped with an interferential filter. The optical head, comprising the lighting system and the catoptric collecting optics, is placed in a 45°/0° illumination/observation geometry and moves with a step of 250 μm and speed of 500 mm/s. During the scanning, the autofocus system maintains the optimal target-lens distance, thanks to a high-speed triangulation distance metre and a custom-made control software. The result is a set of perfectly superimposing monochromatic images free from aberrations and metrically correct. The 16 monochrome images in the Vis spectral range are combined with a custom-made software, using the standard CIE D65 illuminant and the 2° observer, to obtain the RGB image (i.e., colour image) of the scanned surface.

In this work, images in the NIR spectral range were post-processed with a custom-made software to produce false colour (FC) images [22] with the aim of enhancing the visualization of the details of interest. An infrared image from the multispectral cube was combined with the red and green images taken from the RGB image. In this manner, the traditional NRG → RGB mapping is obtained (namely, N → R, R → G, and G → B), with “R”, “G”, and “B” being the red, the green, and the blue channels, respectively, and “N” being the near-infrared spectral band.

Spectral Correlation Mapping (SCM) was also performed on the spectral data acquired with Vis-NIR scanning. SCM represents an improvement of SAM (Spectral Angle Mapper [23]) and is based on similarities between spectral images and a reference spectrum [24]. This mapping method relies on the Spectral Correlation Function (SCF) algorithm, allowing for the identification of differences between data cubes.

FORS was performed to characterize the pigments with a higher spectral resolution (1 nm) with respect to multispectral reflectography. Two commercial (Zeiss) spectrometers, MCS (Multi-Channel Spectrometer) 521 VIS NIR-E and MCS 511 NIR 1.7 were used in conjunction to cover the 300–1700 nm spectral range, with a tungsten-halogen (CLH500) lamp as a light source. The signal is transmitted through optical fibre bundles, bringing the light to the surface of the object, and collecting the light reflected to the detectors. The bundles are connected to a hemispherical probe with 45°/0° illumination/observation geometry to match the same illumination/detection conditions of the multispectral scanner. Proper adjustment procedure was followed before each set of acquisitions by measuring a certified white 100% reflectance reference standard (Spectralon) and performing background noise detection. The diameter of the measured spot was approximately 3 mm, with each spectrum averaged over nine acquisitions.

### 2.3. Laser Scanning Microprofilometry

Morphological analysis was carried out by means of a laser scanning micro-profilometer [25] developed at CNR-INO for measuring a wide range of materials and surfaces. A commercial conoprobe (Optimet, Conoprobe 1000) is moved by a scanning device, allowing for measurements on a maximum area of 30 × 30 cm^2^. The profilometer, which has 1 μm axial resolution, 20 μm lateral resolution, and an 8 mm dynamic range, provides faithful, high-resolution topographic maps of the measured surface, which may be displayed either as a 3D model or as an image. The latter may be further processed through the application of digital filters and rendering techniques to enhance micrometric details and improve their readability.

### 2.4. Spectral-Domain Optical Coherence Tomography (Sd-OCT)

Cross-sectional analysis was performed with a commercial OCT device, Thorlabs Telesto-II, equipped with a superluminescent diode (central wavelength: 1300 nm, bandwidth: about 100 nm) with an axial resolution of 5.5 μm in air, and lateral resolution of 13 μm. The maximum field of view (FOV) is 10.0 × 10.0 mm^2^, with 3.5 mm imaging depth. The detector consists of a spectrograph equipped with a diffraction grating and a fast camera. The system is controlled via 64-bit software preinstalled on a high-performance computer. The 3D scanning path probe with integrated video camera allows for high-speed imaging (76 kHz) for rapid volume acquisition and live display. The sample stage provides XY translation and rotation of the sample along with axial travel of the probe.

### 2.5. Radiocarbon Dating by Accelerator Mass Spectrometry (AMS)

Radiocarbon dating was performed by Accelerator Mass Spectrometry using the dedicated beam line of the HVEE 3 MV Tandem accelerator installed at the INFN-LABEC laboratory in Florence [26,27]. Before the measurements, the sample was prepared using the Acid–Base–Acid procedure [28] to remove the possible natural contaminations from exogenous carbon. To convert the sample to graphite, i.e., the chemical form suitable for the LABEC accelerator ion source, the sample was combusted using an elemental analyser (Thermo Flash EA 1112) and the produced CO_2_ was collected into the graphitization line for the production of elemental carbon through the Bosch reaction [29,30]. The correction for the background and the isotopic fractionation were obtained by measuring the ^14^C concentration in blank samples and the ^13^C/^12^C isotopic ratio along the accelerator beam line, respectively. Finally, the ^14^C/^12^C isotopic ratios were normalized using NIST Oxalic Acid II standard samples. The calibrated age ranges were obtained using the OxCal v.4.3.3 software [31], taking as a reference the IntCal20 [32] calibration curve.

## 3. Results

### 3.1. Multispectral Reflectography and Fibre-Optics Reflectance Spectroscopy (FORS)

Multispectral analysis allowed for the determination of a complete set of spectral endmembers, namely a subset of spectra in the image cube corresponding to the most spectrally diverse pixels, representing the signatures of the different materials in the painting. Spectral endmembers were collected all over the painting surface based on differences highlighted by the false colour (FC) [22] images. Most notable colour changes were observed in the lower half of the painting, as shown in Figure 1 (FC images of the entire painted surface can be found in the Supplementary Material Figure S1). Specifically, the red areas in the RGB image (interpreted as the hoop earring and the neckline of the dress) appear yellow in the FC image at 950 nm, and become gradually green at longer wavelengths. The same happens for the red lips of the woman and in the upper background of the painting (not visible in the details in Figure 1). The dark-bluish background at the bottom-left of her neck, as well as distinct grains near the yellowish pearls in her hair, display a purple-pink hue in all the FC images. A detail emerges in the bottom-right red areas in the FC images between 1230 and 2200 nm, namely an oblique line gradually turning bright orange, possibly belonging to the decoration of the dress. The same orange hue can be observed on the earring in the woman’s right ear. In the dark area below her neck, well-defined grains can be distinguished from the surroundings, as they turn bright red in the FC images, especially at 1500 nm.

**Figure 2:**
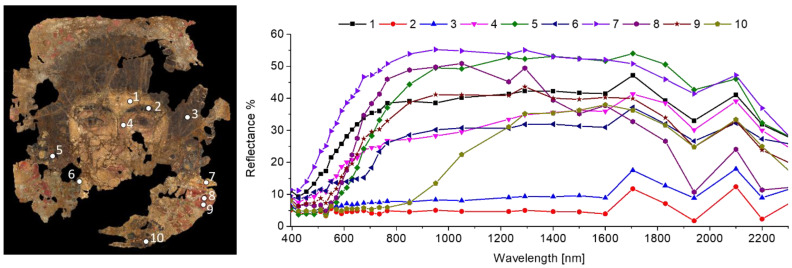
Colour image of the Fayum mummy portrait (on the **left**) showing the representative points where the spectra of the 10 endmembers (on the **right**) were extracted from the multispectral data-cube for spectral correlation analysis.

The spectral analysis found ten endmembers adequately describing the portrait’s surface, whose spectra are reported in Figure 2 with their representative location on the painting surface.

Spectral Correlation Mapping (SCM), based on the comparison of the spectral images with the endmembers, was performed to produce the spectral maps showing the distribution of the pigments, or pigment mixtures, all over the painted surface. The distribution maps are displayed in white, meaning maximum similarity with the reference endmember, and then superimposed on the RGB image to facilitate their reading (all SCM images are reported in the Supplementary Material—Figures S2 and S3). To obtain a thorough spectral characterization, several pointwise FORS measurements were collected within each region defined by SCM. Pigments were identified by comparison with spectral databases (CNR-INO), considering the possible effects of paint layering and pigment mixtures, as well as by verifying the accordance with compositional information on other portraits reported in the literature [7,16,33].

**Figure 3:**
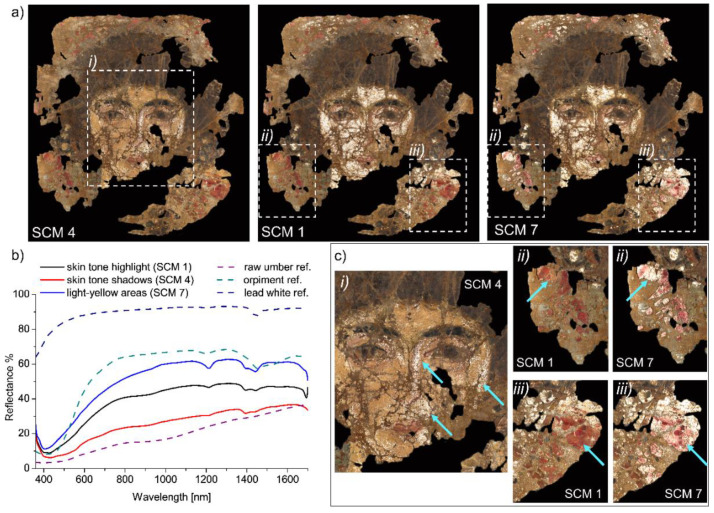
(**a**) SCM 4, 1, and 7 with significant regions (*i*, *ii*, and *iii*) highlighted by the white dashed rectangles and magnified in c. Reference spectra and averaged FORS spectra (**b**) of the skin tone (highlight and shadows) and the light-yellow areas acquired in correspondence of the spectral correlation maps. (**c**) Magnified details of SCM 4, 1, and 7. The light blue arrows point out the differences between the three distributions.

The endmembers of white-yellow, brown, and red areas allowed for the distinction of partially overlapping distributions of pigments, thus suggesting the use of mixtures or superimposed paint layers. Specifically, two slightly different distributions were obtained for the skin tone, one covering mostly the highlights—SCM 1, the other the shadows—SCM 4 (Figure 3a, left and middle, respectively). The third partially overlapping distribution, SCM 7 (Figure 3a, right), revealed a spectral similarity between the flesh tone and the light-yellow areas, i.e., the upper background and few details in the woman’s jewellery. Significant details of the SCM distributions are magnified in Figure 3c (regions *i*, *ii*, and *iii*). FORS analysis of the examined areas is reported together with the reference spectra of raw umber (brown iron-oxide) [34], orpiment (arsenic sulphide, As_2_S_3_ [35]), and lead white [7] (Figure 3b), which are characterized by spectral features compatible with those of the endmembers. Lead white was likely used in varying amounts to light up the hue, as confirmed by the distinctive absorption band at ~1445 nm [7]. The inflection point was at 580–590 nm in the spectrum of SCM 4 is consistent with vermillion (HgS), which was probably added to the mixture to enhance the warm hue of the skin tone around the eyes and the nose.

**Figure 4:**
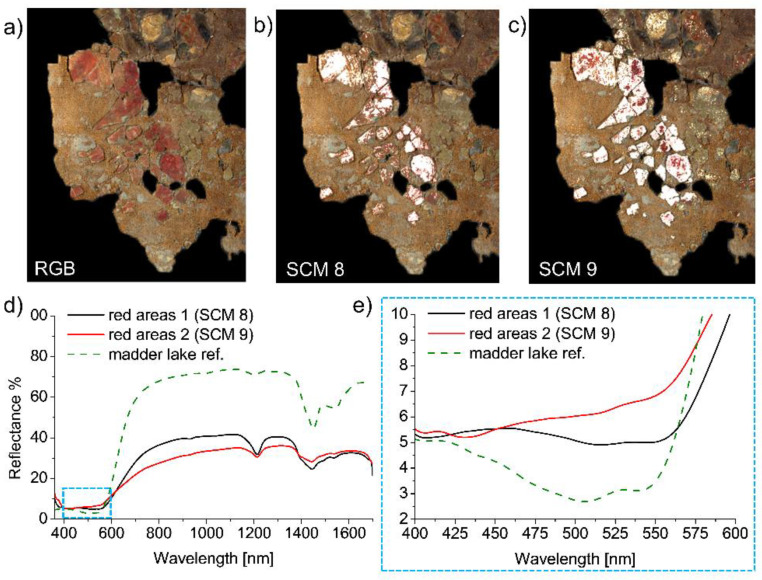
Spectral analysis of the orange decorations and the bluish background: (**a**) RGB image highlighting the relevant region revealed by SCM 8 (**b**) and 9 (**c**). (**d**) FORS spectra of the red pigments in comparison with the reference spectrum of madder lake. The dashed light-blue rectangle evidences the range of 400–600 nm, which is magnified (**e**) to make visible the characteristic features of the pigments.

**Figure 5:**
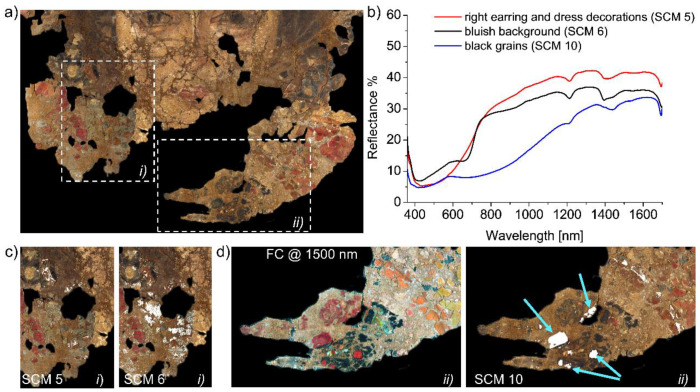
Spectral analysis decoration areas identified by SCM 5, 6, and 10: (**a**) RGB image highlighting the relevant regions *i* and *ii*, magnified in (**c**,**d**); (**b**) FORS spectra of endmembers 5, 6, and 10; (**c**) Comparison of SCM 5 and 6 in region *i*. (**d**) Region *ii* in the FC image and SCM 10 showing the black grains, pointed out by the light blue arrows.

Spectral correlation mapping of the red areas suggests that more than one red pigment was used for the decoration contouring the woman’s face (RGB image of a relevant detail in Figure 4a). Specifically, SCM 8 (Figure 4b) highlights the presence of a red pigment in only some confined areas of those appearing red in the visible light. On the other hand, SCM 9 (Figure 4c) covers a larger amount of red surface, but still excludes some spots. The FORS analysis (Figure 4d,e) shows that endmember 8 is characterized by an absorption band structured into two sub-bands at 500–560 nm, a shoulder at 480–500 nm, a peak of reflectance at about 420 nm, and a sharp increase in reflectance at about 600 nm into the NIR. These spectral features are consistent with madder lake [36,37,38]. SCM 9 has a spectral behaviour similar to SCM 8, but with slight differences (namely a weaker absorption at 500–560 nm, and the absence of the shoulder at 480–500 nm), suggesting the presence of other historically compatible pigments, e.g., cinnabar (characterized by a flat absorption between ~400 and ~570 nm) [39] and orpiment, in accordance with the already mentioned SCM 1 and 7. The use of this mixture would also explain the warm hue of the considered area. Spectral analysis of the orange decorations and bluish background (Figure 5a–d) allowed for a tentative identification of the pigments used. Regions *i* and *ii* in Figure 5a highlight significant details of SCM 5, 6, and 10 Figure 4c,d. Spectral features of SCM 5 (red curve in Figure 4b) suggest the presence of orpiment (see reference spectrum in Figure 3b), possibly used in a blend or a layered structure with a red pigment, such as the already identified vermillion, to obtain the orange hue. The spectrum of the bluish background (black curve in Figure 4b) indicates the possible use of a mixture of blue-green pigments, having a small transition around 575 nm and another inflection point at ~710 nm, which are compatible with green earth and ultramarine [40], respectively. Finally, the black grains turning red in the FC at 1500 nm (Figure 4d, left) stand out in SCM 10 (Figure 4d, right). The latter displays a number of spots, whose FORS spectra have an inflection point at around 480 nm, characteristic of lead-tin yellow and orpiment [35]; the same spectra’s slow rise at 800–1300 nm matches a Cu-containing green, such as malachite [40]. This increase of the reflectance in the NIR is consistent with the FC colour images.

Spectral mapping of the brown areas (see Figure S2 in the Supplementary Material) shows two partially overlapping distributions (SCM 2 and 3), both covering the hair area, whereas only one of them (SCM 2) includes the eyes and the dress collar. The corresponding FORS spectra showed similar behaviour for both distributions, namely low intensity reflectance values over the entire acquisition range, which can be attributed to the presence of charcoal black. The main difference between the two spectra was the presence of an inflection point at 550 nm, visible only in SCM 2, which is consistent with iron oxide pigments (possibly umber and yellow ochre [35]) mixed with other pigments.

### 3.2. Laser Scanning Microprofilometry

**Figure 6:**
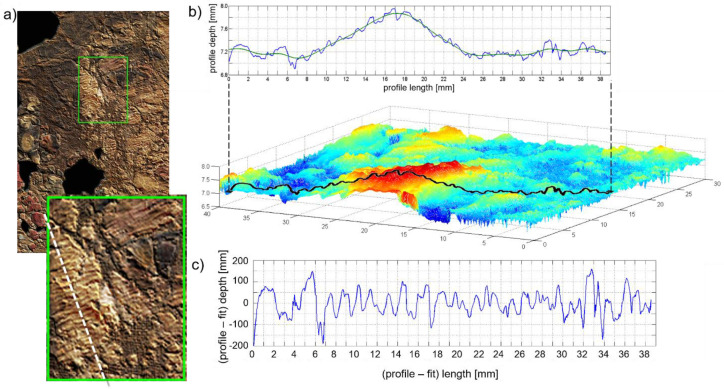
(**a**) Detail of the topographic map superimposed on the RGB image; the white dashed line indicates the location of one of the profiles extracted for the measurement of the grooves. The topographic map (**b**) provided the profile of the surface, from which the surface shape was subtracted (**c**).

The micrometric examination of the surface topography allowed for the visualization of 3D features related to the different tools used to spread the paint, namely strokes of a fine-hair brush that are visible on the red-yellow background, and sequences of grooves, observable in the woman’s skin tone, eyes, nose, and mouth. The latter marks can be easily ascribed to a small spatula, such as a heated cauterium, which is typical of the encaustic technique and has already been identified in other portraits [7,16]. The scanning of the entire surface was performed with a sampling step of 100 μm (see Figure S4 in the Supplementary Material for the topographic map of the entire surface). Few smaller regions, such as the sequence of well-defined grooves on the woman’s right cheekbone (Figure 6a), were measured with a 50 μm sampling step. Surface profiles were extracted from the topographic map (Figure 6b) to measure the size of the marks after the subtraction of the surface shape by spline interpolation (green line in Figure 6b). The difference in the grooves size resulted in a range of 0.5–1 mm, which is likely due to a successive application of the cauterium on the same area.

### 3.3. Spectral-Domain Optical Coherence Tomography

**Figure 7:**
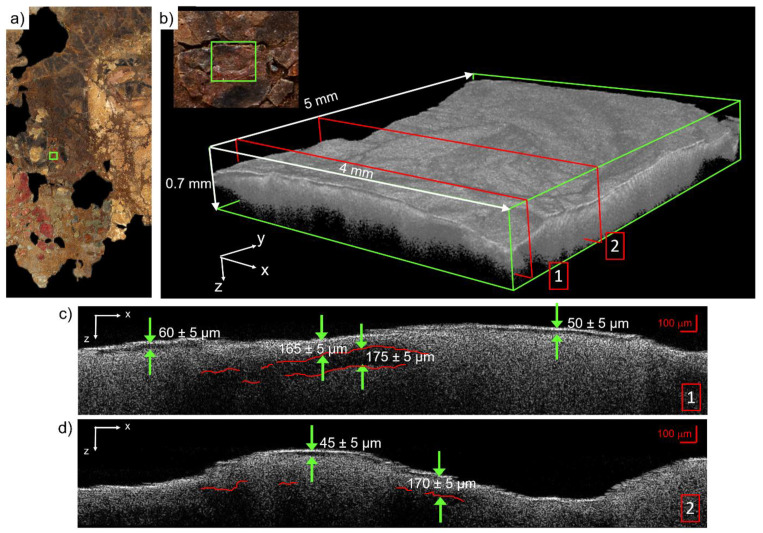
OCT analysis performed on the woman’s right earring: (**a**) RGB image detail showing the acquisition area (green rectangle); (**b**) Tomocube (5 × 4 × 0.7 mm^3^, voxel size 3.5 μm^3^) highlighting the position of the z–x sections (red slices 1 and 2) reported in (**c**) and (**d**), respectively. The green arrows point out the distance between different material layers for thickness measurements.

Cross-sectional analysis was performed in several regions of the painted surface to verify the presence of paint layering, as it was suggested by the spectral results (Section 3.1). The presence of superimposed layers was confirmed by OCT, which also indicated discontinuities possibly due to the loss of the superficial material. As an example, we reported the OCT results on the right earring, in which the possible presence of orpiment and vermillion was identified (see SCM 5, Figure 5). The acquisition area is indicated by a green square in the RGB image and is shown in magnified detail in Figure 7a,b, respectively. The tomocube (5 × 4 × 0.7 mm^3^, voxel size 3.5 μm^3^) in Figure 7b shows the surface micrometric morphology and allows for the visualization of the multi-layered structure of the painting. The red slices point out the position of the y-z sections extracted from the tomocube for the cross-sectional measurement (Sections 1 and 2 in Figure 7c,d, respectively). The thickness of the superficial layer ranges from 45 to 60 μm ± 5 μm, depending on the site. In Section 1, the lack of signal by about 175 μm in depth (delineated in red) is possibly attributed to a localized discontinuity between the paint layer and another underlying material. The latter could be either the canvas support or another pigment layer characterized by diffusing properties at 1300 nm (wavelength of the radiation probe), which are similar to those of the upper layer. These results are consistent with the previous hypothesis of the presence of at least two superimposed layers containing orpiment and vermillion, with thicknesses of 40–65 μm and 160–175 μm, respectively.

### 3.4. Radiocarbon Dating

Dating was performed on a detached small fragment of canvas (weight ~100 mg), which could not be repositioned on the portrait. Before any cleaning treatment, we selected only the fragment’s fibres that did not present any macroscopic possible contaminations, such as pigments and/or primer traces. A mass of about 27 mg was thus recovered and further processed before the combustion and graphitization phases. Observation under an optical microscope did not reveal any trace of material possibly related to anthropogenic contamination. The sample was then treated to remove natural contaminations, such as secondary carbonates and humic acids. After the chemical procedure, two independent fractions of the cleaned sample were combusted and graphitized to check for statistical consistency.

Measurement results are shown in Table 1. The best estimation of the concentration of ^14^C and of the corresponding conventional radiocarbon age, expressed in pMC and in years BP, respectively, result from the weighted average of the two measured fractions.

**Figure 8:**
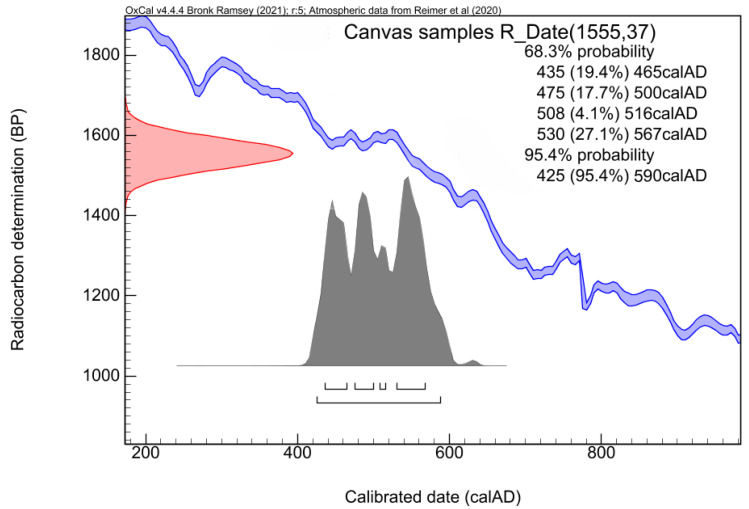
Calibration of the conventional radiocarbon age (1555 ± 37 years BP) measured for the canvas fragment: the IntCal20 calibration curve (conventional radiocarbon age vs. calendar date) is represented in blue; the measured conventional radiocarbon age is treated as a normal-distributed variable and represented in red on the y axis. The grey area on the x-axis represents the distribution of the calibrated age. The calibrated age intervals, calculated at a 68% and 95% level of probability, are reported in details in the graph.

After calibrating the result of the conventional radiocarbon age measurement, the canvas’ origin was set to date back to 425–590 AD and 435–570 AD, with a probability of 95% and 68%, respectively. In Figure 8, the detailed calibration graph is also reported. The calibration curve (conventional radiocarbon age vs. calendar age) with its uncertainty is shown in blue; the measured radiocarbon age, treated as a normally distributed random variable, is displayed in red on the y axis. The calibration result is reported in grey on the *x* axis as posterior probability distribution. The ranges of the calibrated age are reported in detail, both at 68% to 95% level of probability.

## 4. Discussion and Conclusions

The results reported in this study raise interesting considerations on the funerary portrait tradition in Egypt under the Roman Empire. The spectral analysis revealed the presence of pigments already identified in other portraits and historically compatible with the artistic production of that time. In specific, the spectral analysis of the skin tone, the upper background, and a few details in the woman’s jewellery suggests the presence of raw umber, orpiment, lead white, and vermillion, possibly applied in a mixture of different amounts depending on the area. Most of the red decoration contouring the woman’s face shows spectral similarities with cinnabar and orpiment, while madder lake was identified only in some confined areas. Orpiment was possibly used in a blend or a layered structure, with vermillion for the orange decorations. The analysis of the bluish background suggests the presence of blue-green pigments, namely green earth and ultramarine. The spectral behaviour of the black grains below the woman’s neck is consistent with a mixture of lead-tin yellow, orpiment, and malachite. Finally, charcoal black and iron oxide pigments (possibly umber and yellow ochre) were identified in the brown area corresponding to the woman’s hair.

The morphological analysis of the grooves on the surface was attributed to the tools used for the application of the encaustic paint, which is also consistent with the other portraits studied so far. However, the dating provided by ^14^C-AMS indicates that the portrait analysed here was realized between the 5th and 6th centuries AD, i.e., almost two centuries after the production period of the Fayum corpus previously assessed by archaeological and stylistic studies. The analysed painting would therefore date back to the end of the Roman occupation (30 BC–641 AD), which was marked by violent religious and political controversies, riots, and invasions. The identified age range for the analysed portrait corresponds to the reign of a series of Byzantine emperors, ranging from Theodosius II (408–450 AD) to Justinian (527–565 AD), who was the author of the ambitious but only partly realized renovatio imperii, or “restoration of the Empire”. The portraits’ funerary practice would therefore have remained in use in Egypt even in a time of unrest and critical socio-economic changes. This transition also involved the artistic field, in which the Greek naturalistic style was gradually replaced by a more symbolic approach, typical of the Paleo-Christian iconographic language, leading to a new aesthetic defined by its salient “abstract”, or anti-naturalistic character. The slightly flattened and stylized features of the portrayed woman reflect the influence of this new artistic style and recall well-known iconographies dating back to the same period, such as the mosaics in the mausoleum of Galla Placidia and in the Basilica of San Vitale in Ravenna (Italy), realized between 546 and 548 AD. Noteworthy, some scholars have hypothesized a direct stylistic and technical connection between the Fayum portraits, specifically the Antinoite ones, and the oldest known Byzantine icons, dated around the 7th century AD and conserved in the Saint Catherine’s Monastery of Sinai [4,41]. Postponing the dating of the portraits corroborates this hypothesis, thus filling the time gap between the two artistic productions with extremely interesting implications from the historical-artistic point of view. Considerations following the unprecedented dating of the portrait interests also the encaustic technique, which would have been lost at the end of the 3rd century AD [41] and replaced by the oil-paint technique. Therefore, the analysed portrait would be one of the latest encaustic paintings ever studied, especially considering that the date provided by AMS refers to the canvas support, which is likely antecedent to the time the painting was realized.

## Figures and Tables

**Table 1:** Results of the radiocarbon measurements of the canvas fragment.

	^14^C Conc. (pMC)	T_RC_ (Years BP)	Calibrated Age (68% Prob.)	Calibrated Age (95% Prob.)
Canvas samples	82.40 ± 0.38	1555 ± 37	435–570 AD	425–590 AD

## Supplementary Materials

The following are available online, Figure S1: False colour imaging, Figures S2 and S3: Spectral correlation mapping, Figure S4: Topographic map.
