# Correction: Dal Fovo et al. Multi-Analytical Characterization and Radiocarbon Dating of a Roman Egyptian Mummy Portrait. *Molecules* 2021, *26*, 5268

**DOI:** 10.3390/molecules27123822

**Published:** 2022-06-14

**Authors:** Alice Dal Fovo, Mariaelena Fedi, Gaia Federico, Lucia Liccioli, Serena Barone, Raffaella Fontana

**Affiliations:** 1 National Research Council—National Institute of Optics, Largo E. Fermi 6, 50125 Firenze, Italy; raffaella.fontana@ino.cnr.it; 2 National Institute of Nuclear Physics, Via Bruno Rossi 1, Sesto Fiorentino, 50019 Firenze, Italy; fedi@fi.infn.it (M.F.); liccioli@fi.infn.it (L.L.); serena.barone@fi.infn.it (S.B.); 3 OPD-Scuola di Alta Formazione e Studio, Via Alfani 78, 50121 Firenze, Italy; gaiafederico91@gmail.com

Due to the fact that the policy regarding the publication of images from the collection of the Papyrological Institute, the owner of the object under study, changed when the article was already in publication, the authors would like to make the following corrections to this paper [1] published in *Molecules*. Details of the modifications are listed as follows to help readers to follow the updates:

## Error in Figure

In the original publication, there was a figure (Figure 1) whose publication was not approved by the Papyrological Institute. Figure 1 has now been removed, and the following figures were renumbered accordingly.

## Change in the Affiliation

In the published publication, there was an error regarding the affiliation for “**Gaia Federico**”. 

The original affiliation was: “Opificio delle Pietre Dure, V. le Filippo Strozzi 1, 50129 Firenze, Italy; gaiafederico91@gmail.com”.

The correct afflicatioin should be “OPD-Scuola di Alta Formazione e Studio, Via Alfani 78, 50121 Firenze, Italy; gaiafederico91@gmail.com”. 

## Missing Acknowledgment

In the original publication, “The object analyzed in this study is currently under investigation at the G. Vitelli Papyrological Institute of Florence” was not cited. The citation has now been inserted in the Acknowledgments, and should read:

“Marco Ciatti, conservators Patrizia Riitano, Lucia Bresci, and Licia Triolo from Opificio delle Pietre Dure, and Daniela Manetti and Francesca Maltomini from the G. Vitelli Papyrological Institute of Florence are gratefully acknowledged. The object analyzed in this study is currently under investigation at the G. Vitelli Papyrological Institute of Florence. Measurements discussed in the present paper were performed before the position of L. Liccioli within the IPERION HS project”.

The authors apologize for any inconvenience caused and state that the scientific conclusions are unaffected. The original publication has also been updated.
