# Supplementary material for: Multi-Analytical Characterization and Radiocarbon Dating of a Roman Egyptian Mummy Portrait
Source: Molecules. 2021 Aug 30;26(17):5268. doi: 10.3390/molecules26175268 (PMC8433800; doi:10.3390/molecules26175268)
Supplement: Supplementary file 1 [file molecules-26-05268-s001.zip › molecules-1313669-supplementary.pdf]

*Supplementary Material*

# Multi-Analytical Characterization and Radiocarbon Dating of a Roman Egyptian Mummy PortraitAlice Dal Fovo [1,*], Mariaelena Fedi [2], Gaia Federico [3], Lucia Liccioli [2], Serena Barone [2] and Raffaella Fontana [1]

[1] National Research Council—National Institute of Optics, Largo E. Fermi 6, 50125 Firenze, Italy; raffaella.fontana@ino.cnr.it
[2] National Institute of Nuclear Physics, Via Bruno Rossi 1, Sesto Fiorentino, 50019 Florence, Italy; fedi@fi.infn.it (M.F.); liccioli@fi.infn.it (L.L.); serena.barone@fi.infn.it (S.B.)
[3] Opificio delle Pietre Dure, V. le Filippo Strozzi 1, 50129 Firenze, Italy; gaiafederico91@gmail.com
* Correspondence: alice.dalfovo@ino.cnr.it

## 1. False colour imaging

The monochromatic NIR images were post-processed with a custom-made software using the false colour (FC) method, with the aim of enhancing the visualization of the details of interest to be compared with the photoacoustic image. In this case, the traditional NRG→RGB mapping was used, with "R" being the red, "G" the green and blue "B" channels, respectively, and "N" the near-infrared spectral band.

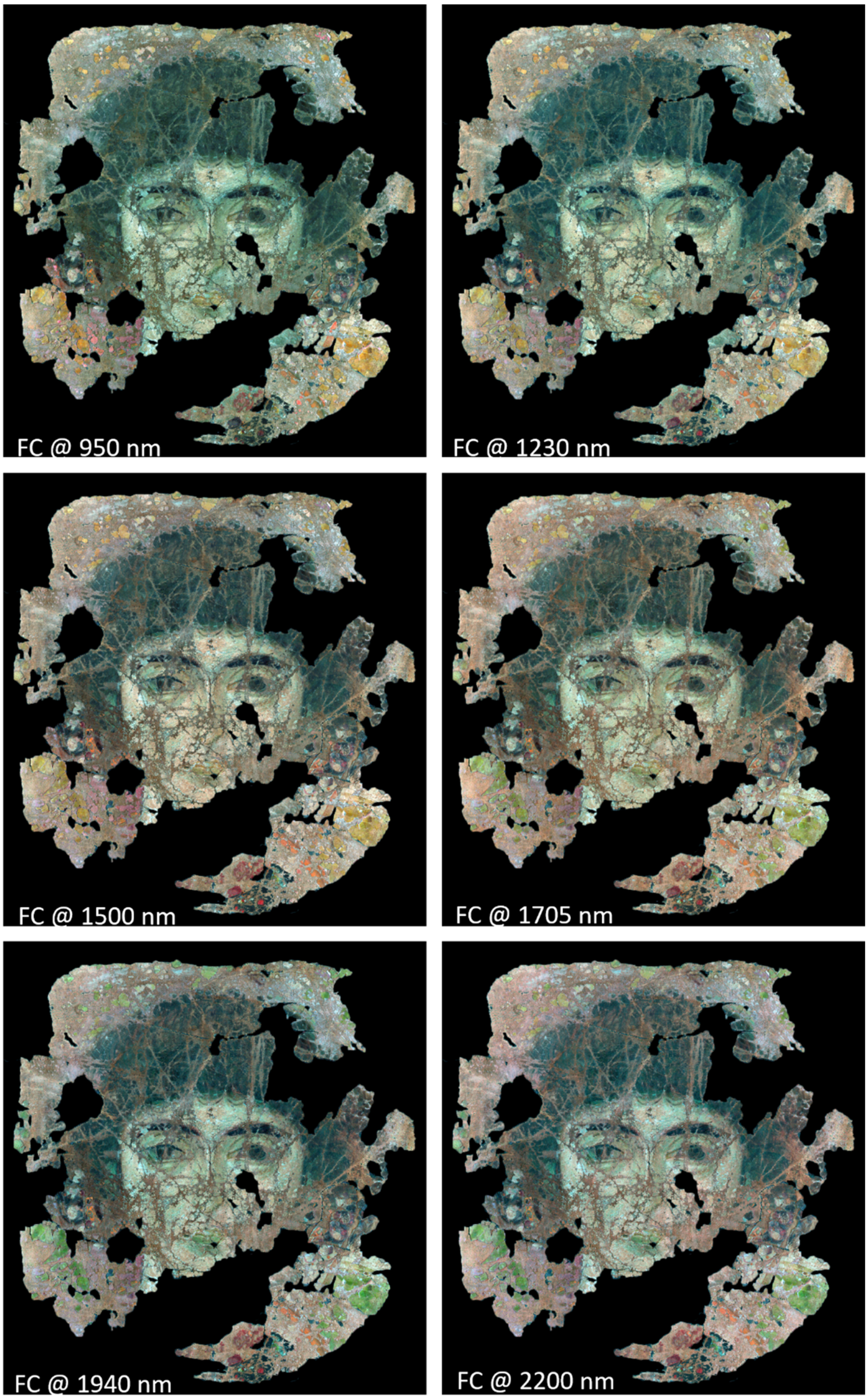


**Figure S1.** False colour images highlighting significant spectral differences in the painting.

## 2. Spectral correlation mapping

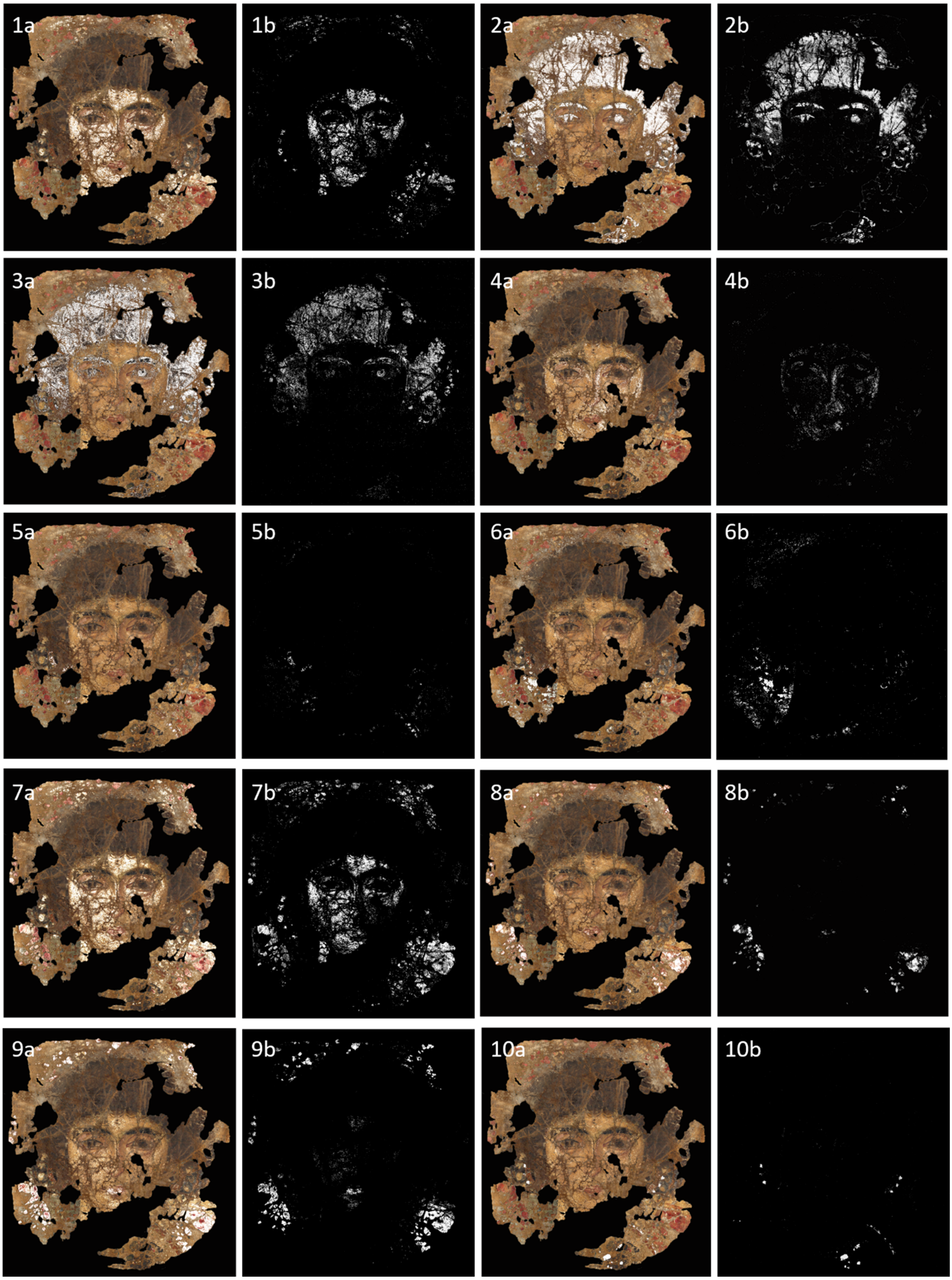


**Figure S2.** Spectral mapping based on ten spectral endmembers (Fig. 1-10) adequately describing the portrait's surface. The distribution maps are displayed in white (b), meaning maximum similarity with the reference endmember, and superimposed on the RGB image to facilitate their reading (a).

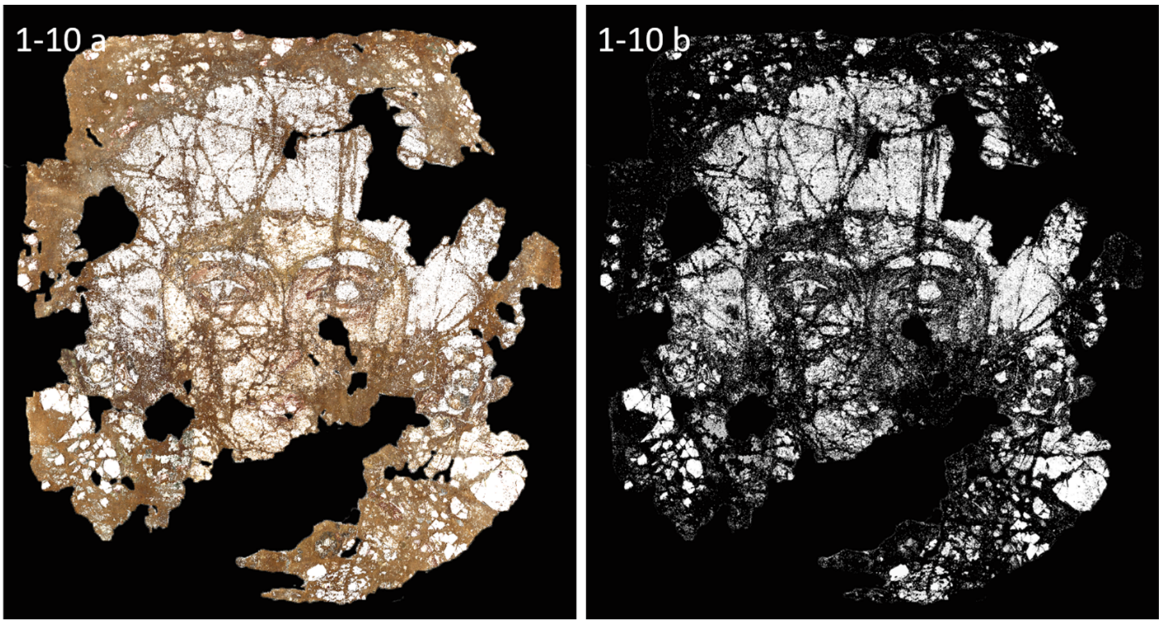


**Figure S3.** 1-10 SCM maps merged together covering the entire painting surface.

## 3. Topographic map

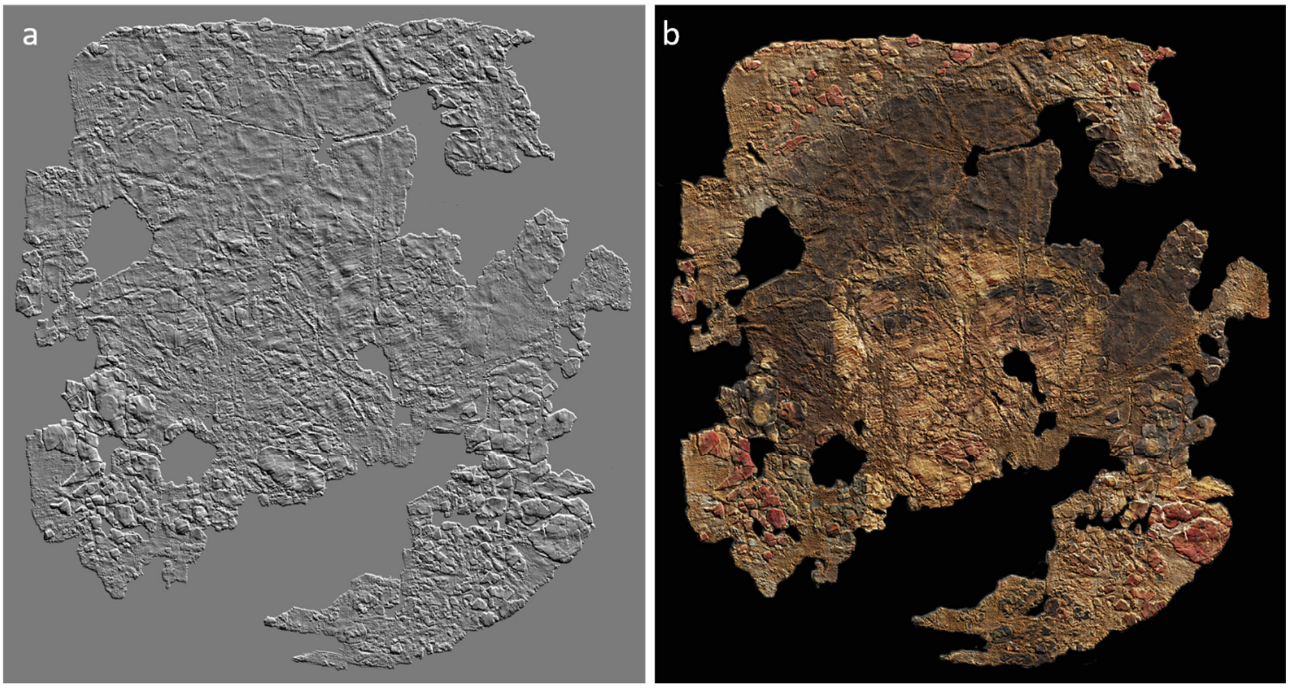


**Figure S4.** High-resolution topographic maps of the measured surface displayed raking light image (a) to enhance micrometric details and improve their readability and superimposed on the RGB image (b) to facilitate the correlation of the micromorphology with the colour information.
